# The critical role of interference control in metaphor comprehension evidenced by the drift–diffusion model

**DOI:** 10.1038/s41598-021-98351-8

**Published:** 2021-09-29

**Authors:** Hee-Dong Yoon, Minho Shin, Hyeon-Ae Jeon

**Affiliations:** 1 Department of Brain and Cognitive Sciences, Daegu Gyeongbuk Institute of Science and Technology (DGIST), Daegu, Korea; 2 Convergence Research Advance Center for Olfaction, Daegu Gyeongbuk Institute of Science and Technology (DGIST), Daegu, Korea; 3 Partner Group of the Max Planck Institute for Human Cognitive and Brain Sciences at the Department of Brain and Cognitive Sciences, DGIST, Daegu, Korea

**Keywords:** Human behaviour, Cognitive control, Language

## Abstract

We address the question of, among several executive functions, which one has a strong influence on metaphor comprehension. To this end, participants took part in a metaphor comprehension task where metaphors had varying levels of familiarity (familiar vs. novel metaphors) with different conditions of context (supporting vs. opposing contexts). We scrutinized each participant’s detailed executive functions using seven neuropsychological tests. More interestingly, we modelled their responses in metaphor comprehension using the drift–diffusion model, in an attempt to provide more systematic accounts of the processes underlying metaphor comprehension. Results showed that there were significant negative correlations between response times in metaphor comprehension and scores of the Controlled Oral Word Association Test (COWAT)-Semantic, suggesting that better performances in comprehending metaphors were strongly associated with better interference control. Using the drift–diffusion model, we found that the familiarity, compared to context, had greater leverage in the decision process for metaphor comprehension. Moreover, individuals with better performance in the COWAT-Semantic test demonstrated higher drift rates. In conclusion, with more fine-grained analysis of the decisions involved in metaphor comprehension using the drift–diffusion model, we argue that interference control plays an important role in processing metaphors.

## Introduction

Time is money. One can easily understand this sentence because time and money have common properties: both are valuable in today’s society in many ways. It does not mean that one can buy food with time, but rather that time is as precious as money to most people. This type of speech, known as a metaphor, is constructed by linking one thing to another that has seemingly different concepts but shares relevant features. In this example, people comprehend the sentence by comparing the topic (the subject of the metaphor: time) and the vehicle (the word used for a metaphor expression: money) of the metaphor based on the ground (the common and relevant features between the topic and vehicle: valuable). Then people select the appropriate meaning of the vehicle and associate it to the topic of the metaphor^1^. Using metaphors enables listeners to easily grasp speakers’ thoughts, but this can be done only when listeners think beyond literal meanings of the vehicle of a metaphor^2^ by selecting the appropriate meaning of the vehicle from several alternatives^3^ to make further inferences about the speaker’s thoughts correctly^2,4^.

### Drift–diffusion models and metaphor comprehension

Previous attempts have been made to examine metaphor processing from various aspects such as the familiarity of a given metaphor, the context in which a metaphor is presented, or individuals’ executive function abilities^5–7^. Researchers have scrutinized behavioral outputs measured by response times (RTs) and accuracies, hoping to reveal underlying processes involved in metaphor comprehension. However, the behavioral measures (i.e., RTs and accuracies) reflect several cognitive processes at the same time^8^, which may inevitably lead to different interpretations of the data. For example, fast stimulus encoding or swift rate of information processing brings about fast RTs. Fast motoric preparation and execution, or less attentive response also affects individuals’ RTs or accuracies. Response biases are one of the critical factors that induces changes in speed of RTs or in accuracy of behavior responses. In other words, various components of response processing seem to be entangled in individuals’ response speed or accuracy, and thus, we need to disentangle them from each other and account for them in detail. To this end, we conducted computational cognitive modeling with the drift–diffusion model^9–11^ to estimate and control for individual differences in metaphor processing with varying levels of familiarity and context.

Computational cognitive modeling has recently gained popularity as a tool to analyze behavioral data since it captures information beyond basic output from participants (e.g., RTs and accuracies), yielding more precise measurement for quantifying cognitive processes of interest^12^. The drift–diffusion model is one such example that models a decision as a process of evidence accumulation, where a decision is assumed to be made after accumulated evidence exceeds a certain threshold^9^. The model additionally introduces a non-decision component, such as encoding time of the stimulus or response execution. Therefore, the drift–diffusion model can extract components that are core to the decision-making process, while excluding decision-unrelated components. Typically, a parameter called drift rate, which indicates the mean speed of evidence accumulation, is employed to study differences between conditions or groups in a task. This parameter accounts for task difficulty such that higher drift rates indicate easier tasks^13^. Compared to the traditional analyses with RTs and accuracies, the analysis with drift–diffusion model parameters provides a more principled approach by decomposing behaviors into various decision-related or non-related components. The model has been used in explaining how decisions are guided by stimulus information and how information is processed over time in human cognition (e.g., attention^14^, working memory^15^, general intelligence^16^, and music cognition^17^). In the present study, we focused on how performance differences in metaphor comprehension could be demonstrated by different parameters of the diffusion model.

### Possible factors influencing metaphor comprehension

Familiar metaphors aid people in understanding one thing with respect to another^18^. Several theories have been proposed in terms of the role of familiarity in metaphor comprehension (for reviews, see^19,20^). According to the graded salience model^21^, figurative meanings of familiar metaphors are salient and can be accessed directly from the mental lexicon without the aid of context. Saliency is determined by several features, such as the metaphor’s familiarity, conventionality, frequency, and the status of preceding context^21^. It is the critical factor that regulates the speed of comprehension; a word with a more salient meaning is processed faster than a word with a less salient meaning^21,22^. Another model—the career of metaphor model^23^—denotes a clear distinction between a familiar metaphor and a novel metaphor by emphasizing the importance of repetition. In general, retrieving or inferring figurative meanings from novel metaphors takes relatively longer than familiar metaphors^24,25^ or literal expressions^2,26–28^, which is due to an increased use of mental resources to make new metaphorical interpretations with the novel expression^6,27,29–33^. However, after being used repeatedly, a novel metaphor also becomes a familiar metaphor so that one can retrieve its figurative meaning fast^23^. To summarize, different levels of familiarity of a metaphor seem to be the crucial factor that deploys varying levels of processing demands in comprehension.

Besides familiarity, context is also an influential factor in metaphor comprehension (for reviews, see^19,20^). Preceding context helps a better understanding of metaphors when it provides sufficient ground that links the topic and vehicle of a metaphor^34,35^. Appropriate contextual information facilitates metaphor comprehension, aiding in the selection of a suitable meaning for the word used for metaphoric expression (vehicle)^36^. In line with this, the prior decision model^37^ suggests that prior information (i.e., the context) guides meaning selection so that people comprehend metaphorical words as quickly as literal words when presented with appropriate context^38–40^. In the same vein, preceding context with relevant information that matches attributes of the vehicle improves metaphor comprehension effectively^20^. For instance, when participants judged whether a metaphorical sentence was true or false, they spent less time on metaphors with appropriate contextual information than with unrelated context^41^. Taken together, supporting and appropriate contexts seem to facilitate metaphor comprehension.

Another factor which has a profound impact on metaphor processing is individuals’ capacity in executive functions^42,43^. Executive functions have been known to comprise several abilities, and they operate as entities, not as a whole^44^. For example, Miyake et al.^44^ made efforts to stipulate to what extent three executive functions (i.e., working memory, cognitive flexibility, and inhibition) share the same underlying mechanism. Resultingly, despite being moderately related to each other, they turned out to be separable and to contribute dissimilarly to individuals’ performances. Accordingly, it is critical to foreground each executive function separately in terms of metaphor comprehension. There have been several attempts to investigate the process of metaphor comprehension together with working memory^34,45–47^, cognitive flexibility^44,48–50^, and inhibition^30,51,52^. For instance, people with a high capacity of cognitive control showed shorter reading times during metaphor comprehension when presented with a prior context^42^. One study suggested that working memory is essential for automatic metaphor processing by showing that individuals with high working memory yielded a smaller metaphor interference effect than those with low working memory^53^. Another study showed that good inhibitory control supported accurate metaphor processing compared to bad inhibitory control^46^. A study of patients with schizophrenia, who are known to be impaired in executive functions^54^, also demonstrated difficulties in processing metaphors^55^. These findings suggest that executive functions are the key factors that contribute critically to processing metaphors.

Inhibition, one of the executive functions, plays a crucial role in metaphor comprehension^18^. One type of inhibition^56^ in particular, namely interference control, has been closely investigated with respect to metaphor comprehension. During metaphor processing we select semantically proper meanings or features of a metaphor vehicle while suppressing frequently used meanings^57^. For instance, to comprehend the metaphor “Those fighters are lions,” one has to retrieve general attributes of the vehicle “lions” (i.e., brave, strong or fierce) instead of its frequently retrieved meaning (i.e., a large tawny-colored cat that lives in prides, found in Africa and north-western India)^58^. By inhibiting irrelevant meanings of a metaphorical expression (i.e., vehicle), one can successfully infer the designated meaning of a metaphor^30,36,46,51,52,57^. Therefore, metaphor comprehension is more likely to be successful when an infrequently used but contextually more adequate meaning of a vehicle is selectively processed instead of its prepotent meaning, and to this end, interference control would inevitably be involved.

### Hypothesis

In the present study, we investigated how familiarity and context would influence metaphor comprehension and scrutinized which of the executive functions was mostly intertwined with it. More specifically, we focused on whether performance differences in metaphor comprehension, modulated by varying levels of familiarity and context, would be demonstrated by different parameters of the drift–diffusion model (interference control as a between-participant factor, and familiarity and context as within-participant factors). We hypothesized that the familiarity of a metaphor, the supportiveness of a context, and individuals’ interference control would exert a significant influence on the process of metaphor comprehension. We expected that individuals with good interference control would perform better in a metaphor comprehension task and show higher drift rates. In addition, we expected that familiar metaphors and supporting context would aid in metaphor comprehension, such that participants would exhibit higher drift rates in these conditions.

## Results

Participants were engaged in a metaphor comprehension task. On each trial, they first read a context sentence, either supporting (SC) or opposing (OC), followed by either a familiar metaphor (FM) or a novel metaphor (NM). Participants were required to judge whether the two consecutive sentences made sense or not as quickly as possible. Accordingly, there were four experimental conditions: a supporting context paired with a familiar metaphor (SC–FM) or a novel metaphor (SC–NM), and an opposing context paired with a familiar metaphor (OC–FM) or a novel metaphor (OC–NM).

### RTs and accuracies in metaphor comprehension

**Figure 1:**
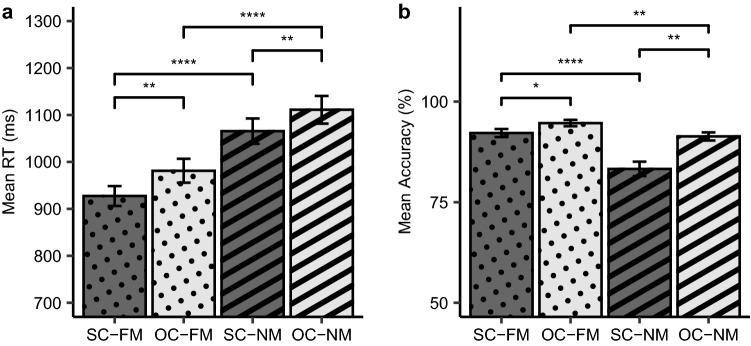
Significant differences in RTs and accuracies across conditions. (**a**) Bar plot depicts the average of the participants’ mean RTs in each condition. The x-axis denotes the four conditions of the metaphor comprehension task, while the y-axis shows the RTs in milliseconds (ms). There were significant RT differences in FAMILIARITY (i.e., SC–FM vs. SC–NM, OC–FM vs. OC–NM) and COTEXT (i.e., SC–FM vs. OC–FM, SC–NM vs. OC–NM). (**b**) Bar plot depicts the average accuracies in each condition. The x-axis denotes the four conditions of the metaphor comprehension task, while the y-axis shows the accuracies (% correct response). There were significant accuracy differences in FAMILIARITY (i.e., SC–FM vs. SC–NM, OC–FM vs. OC–NM) as well as in CONTEXT (i.e., SC–FM vs. OC–FM, SC–NM vs. OC–NM). A significant interaction of accuracy across conditions was also demonstrated. Error bars indicate the standard error of the means. Bar colors represent the supportiveness of the context sentences (dark gray: SC, light gray: OC), and patterns of the bar illustrate the familiarity of the metaphor (dots: FM, stripes: NM). *SC–FM* supporting context with familiar metaphor, *OC–FM* opposing context with familiar metaphor, *SC–NM* supporting context with novel metaphor, *OC–NM* opposing context with novel metaphor. *p < 0.05; **p < 0.01; ****p < 0.0001, Bonferroni corrected.

To probe the effect of familiarity and context during metaphor processing, we performed two-way repeated measures analyses of variance (ANOVA) with factors FAMILIARITY (FM and NM) and CONTEXT (SC and OC) using the RTs and accuracies of the metaphor comprehension task. The bar plot of the RT data for the four conditions are illustrated in Fig. 1a. We found significant main effects in both FAMILIARITY [*F* (1, 37) = 124.48, *p* < 0.001] and CONTEXT [*F* (1, 37) = 16.48, *p* < 0.001], but there was no interaction [*F* (1, 37) = 0.19, *p* = 0.67]. In terms of the familiarity, RTs in FMs (SC–FM and OC–FM: mean = 954.30 ms; standard error of the means (SEM) = 23.62) were faster than RTs in NMs (SC–NM and OC–NM: mean = 1088.36 ms; SEM = 28.27). With respect to the context, RTs in SCs (SC–FM and SC–NM: mean = 996.53 ms; SEM = 26.55) were faster than RTs in OCs (OC–FM and OC–NM: mean = 1046.13 ms; SEM = 29.31).

For the accuracy data, we found main effects of FAMILIARITY [*F* (1, 37) = 50.69, *p* < 0.001] and CONTEXT [*F* (1, 37) = 16.14, *p* < 0.001]. We also observed an interaction [*F* (1, 37) = 6.87, *p* = 0.01] between the two factors (Fig. 1b). Participants’ accuracies were higher in OCs than in SCs for both FMs and NMs, but this difference between SCs and OCs was greater in NMs (8.06%) than in FMs (2.46%). Overall, participants responded more accurately in FMs (SC–FM and OC–FM: mean = 93.42%; SEM = 0.91) than NMs (SC–NM and OC–NM: mean = 87.31%; SEM = 1.58) and in OCs (OC–FM and OC–NM: mean = 93.00%; SEM = 0.94) than SCs (SC–FM and SC–NM: mean = 87.73%; SEM = 1.61).

### Significant correlations between RTs from all conditions and the scores from COWAT-Semantic

**Figure 2:**
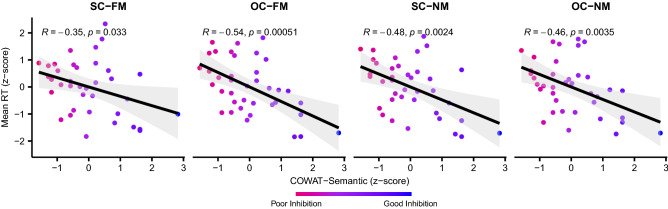
Significant correlations between RTs of the metaphor comprehension task and the scores of COWAT-Semantic. The x-axis indicates the scores of COWAT-Semantic and the y-axis denotes participants’ RTs. Both values were converted to z-scores. Each dot represents an individual’s data, and colors of the dots imply each participant’s level of competence in inhibition. The error bands indicate 95% confidence intervals. There was a tendency for the individuals with good inhibition capabilities (i.e., higher scores in the COWAT-Semantic) to respond faster to metaphor comprehension task in all the conditions compared to individuals with poor inhibition capabilities (i.e., lower scores in the COWAT-Semantic). *SC–FM* supporting context with familiar metaphor, *OC–FM* opposing context with familiar metaphor, *SC–NM* supporting context with novel metaphor, *OC–NM* opposing context with novel metaphor, *COWAT* controlled oral word association test.

Pearson correlation coefficients between the scores of the seven neuropsychological tests (see “Supplementary Materials”) and the RT data from the four conditions (SC–FM, SC–NM, OC–FM, and OC–NM) were computed to examine the roles of different executive functions on metaphor comprehension. Correlations between participants’ RTs from the four conditions and the scores from the seven neuropsychological tests are shown in Table 1. Semantic fluency task of the Controlled Oral Word Association Test (COWAT-Semantic) showed significantly negative correlations with all four conditions (Fig. 2), indicating that the better participants were in the COWAT-Semantic, the shorter RTs were in metaphor comprehension. Additionally, Go/No-Go (GNG) task scores revealed a significantly positive correlation with the RTs in the OC–NM condition.

**Table 1:** Pearson correlation coefficients between the scores of neuropsychological tests and RTs in each condition.

	SC–FM	OC–FM	SC–NM	OC–NM
ANT	− 0.07	0.20	0.16	0.14
COWAT-Semantic	− 0.35*	− 0.54***	− 0.48**	− 0.46**
COWAT-Phonemic	− 0.25	− 0.31	− 0.19	− 0.25
GNG	0.28	0.29	0.27	0.34*
LNST	− 0.09	− 0.03	− 0.08	− 0.11
Stroop	0.19	0.00	0.05	0.12
WCST	− 0.07	0.09	0.09	0.06

*SC–FM* supporting context with familiar metaphor, *OC–FM* opposing context with familiar metaphor, *SC–NM* supporting context with novel metaphor, *OC–NM* opposing context with novel metaphor, *ANT* attention network test, *COWAT* controlled oral word association test, *GNG* go/no-go, *LNST* letter number sequencing task, *WCST* Wisconsin card sorting test.

*p < 0.05.

**p < 0.01.

***p < 0.001.

### Results from the hierarchical drift–diffusion model (HDDM)

To further analyze the decision process during metaphor comprehension, we adopted the drift–diffusion model. We first show that incorporating familiarity, context, and individual differences in executive functions improved the fit of the drift–diffusion model via model comparison. The specific influences of such factors during metaphor comprehension are then investigated.

#### Model comparison

**Figure 3:**
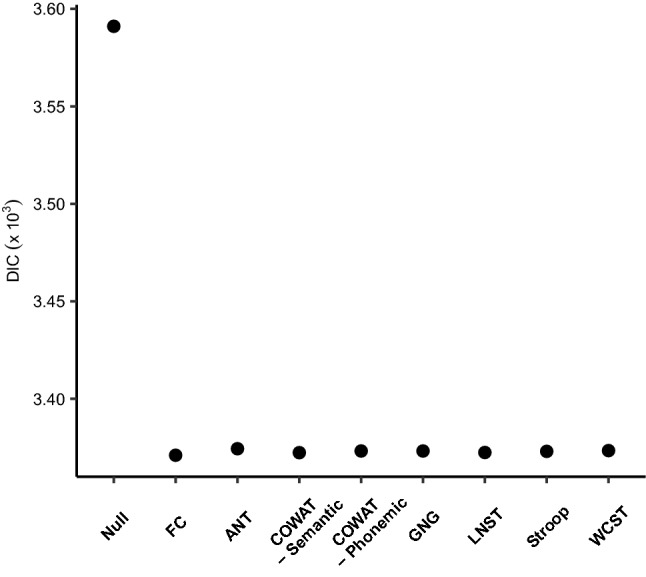
DIC values for nine candidate models with regard to seven neuropsychological test performances. DIC values for the *Null* model, the *FC* model, and seven *Full* models for each neuropsychological test are shown. Here, lower values indicate better fits. The *FC* model and seven *Full* models outperformed the *Null* model, but the differences between the *FC* model and the *Full* models are marginal. Models are displayed in alphabetical order after two base models (*Null* and *FC* model). *ANT* attention network test, *COWAT* controlled oral word association test, *GNG* go/no-go, *LNST* letter number sequencing task, *WCST* Wisconsin card sorting test.

We used deviance information criterion (DIC), which is a well-known measure for model comparison^59^. The DIC value was calculated for nine different models: seven *Full* models for each neuropsychological test, a *Null* model, and an *FC *(*familiarity and context*) model (see “Methods” for the detailed definition of each model)*.* We set the *Null* and *FC* models as criterion for base models and checked whether appraising the four conditions of the metaphor comprehension task or individual performances on various tests showed better fit compared to the two base models. As shown in Fig. 3, all the models outperformed the *Null* model with more than 200 DIC scores. However, DIC scores between seven variant models and the *FC* model were hardly distinguishable. This could indicate that adding terms for individual differences did not make a huge difference in model fits. To further investigate this result, we assessed posterior estimates of decision parameters in seven *Full* models that provide the maximum information compared to two base models (the *Null* and *FC* models).

#### Effects of familiarity and context on drift rate

To investigate whether manipulating familiarity or context has an impact on metaphor comprehension, we analyzed differences in the drift rates between levels of each factor (i.e., FAMILIARITY and CONTEXT). More precisely, we examined differences in the population-level posterior distributions, considering factors within the experimental design for each neuropsychological test.

**Figure 4:**
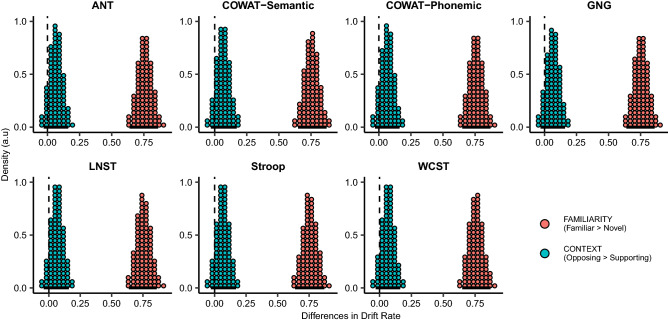
Population-level differences of drift rates with regard to the two factors (FAMILIARITY and CONTEXT). Population-level differences of posterior estimates for drift rates in seven *Full* models are described as quantile dotplots. One hundred dots for each posterior distribution represent quantiles from 0.5 to 99.5%. Therefore, the dots correspond to the posterior probability of whether differences in drift rates between the levels of each factor are positive or negative. In the case of the FAMILIARITY factor, since the difference between FMs and NMs was positive, drift rates were higher when participants were presented with FMs than with NMs (Familiar > Novel), with 100% credibility for all neuropsychological tests. With respect to CONTEXT, even if posterior estimates of drift rates were higher in OCs than in SCs (Opposing > Supporting), 95% HDI included zero in all the tests, meaning that the effect of CONTEXT on the drift rates was statistically insignificant. On the basis of a Bayesian hypothesis testing perspective, a factor has an effect on the drift rate when 95% HDI does not include zero. As more density resides away from zero, the effect of that factor becomes stronger. The black bars under dotplots represent 95% HDIs. *ANT* attention network test, *COWAT* controlled oral word association test, *GNG* go/no-go, *LNST* letter number sequencing task, *WCST* Wisconsin card sorting test, *HDI* highest density interval.

In the FAMILIARITY factor, drift rates in posterior group estimates of FMs were higher than those of NMs for 100% of all the posterior samples across all the neuropsychological tests (Table 2, Fig. 4). On the other hand, posterior estimates of the drift rates in the CONTEXT factor demonstrated that, for all the neuropsychological tests, drift rates for OCs tend to be higher than those for SCs, but all of the 95% highest density intervals (HDIs) contained zero (Table 2, Fig. 4). Usually, a factor is regarded as being influential on drift rates when 95% HDIs do not include zero^60^. As a consequence, we suggest that familiarity provided highly reliable information that affected the decision process of metaphor comprehension, whereas context showed a marginal effect on the process.

**Table 2:** Posterior means and 95% HDIs of drift rates in each factor (FAMILIARITY and CONTEXT) with regard to seven neuropsychological test performances.

	FAMILIARITY (familiar > novel)		CONTEXT (opposing > supporting)	
	Mean	95% HDI	Mean	95% HDI
ANT	0.76	[0.64, 0.86]	0.07	[− 0.03, 0.16]
COWAT-Semantic	0.76	[0.65, 0.87]	0.06	[− 0.03, 0.17]
COWAT-Phonemic	0.76	[0.65, 0.87]	0.07	[− 0.03, 0.16]
GNG	0.75	[0.64, 0.86]	0.06	[− 0.03, 0.16]
LNST	0.76	[0.64, 0.86]	0.06	[− 0.04, 0.16]
Stroop	0.76	[0.64, 0.87]	0.06	[− 0.03, 0.17]
WCST	0.76	[0.65, 0.87]	0.07	[− 0.03, 0.16]

*ANT* attention network test, *COWAT* controlled oral word association test, *GNG* go/no-go, *LNST* letter number sequencing task, *WCST* Wisconsin card sorting test, *HDI* highest density interval.

#### The influence of individual performance in neuropsychological tests on metaphor comprehension

**Figure 5:**
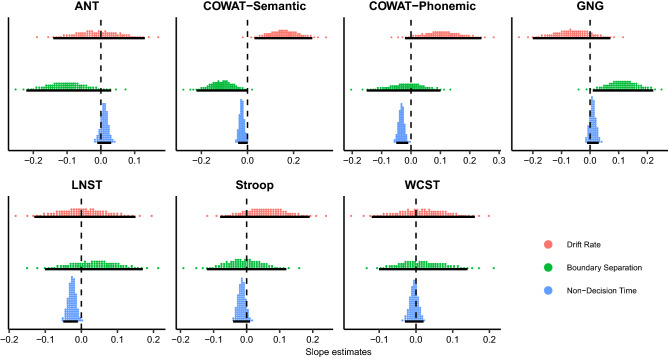
Population-level slope estimates of decision parameters with respect to seven neuropsychological test performances. To illustrate the relationship between participants’ performances in each test and the decision parameters, population-level slope estimates of drift rates, boundary separation, non-decision time are described as quantile dotplots. As 95% HDI of a certain parameter’s estimates (illustrated as black bars) moves away from zero, individuals’ performances in the neuropsychological test are positively or negatively correlated with the decision parameters. The scores in ANT, Stroop task, and WCST were transformed to indicate that higher scores represent better performance. *ANT* attention network test, *COWAT* controlled oral word association test, *GNG* go/no-go, *LNST* letter number sequencing task, *WCST* Wisconsin card sorting test, *HDI* highest density interval.

We assessed how individuals’ executive functions had influence on metaphor comprehension by inspecting 95% HDIs of three varying parameters such as drift rate ($v$), decision boundary ($a$), and non-decision time ($t$) in each neuropsychological test (Table 3). Figure 5 shows a positive linear trend of COWAT-Semantic performance on drift rate, indicating that the better one’s COWAT-Semantic performance, the higher drift rate is observed. A negative correlation was found between the boundary separation and COWAT-Semantic performance, explaining that worse performance in COWAT-Semantic led to a wider decision boundary. Contrarily, a positive relationship was found in GNG such that better performance in GNG corresponded to a wider decision boundary. Non-decision time had negative relationships with performances in COWAT-Semantic, COWAT-Phonemic, and Letter Number Sequencing Task (LNST) in terms of conventional 95% HDIs^60^. This indicates that individuals with worse performance in these tests showed longer non-decision times.

**Table 3:** Posterior means and 95% HDIs of decision parameters with regard to seven neuropsychological test performances.

	Drift rate		Boundary separation		Non-decision time	
	Mean	95% HDI	Mean	95% HDI	Mean	95% HDI
ANT	− 0.01	[− 0.14, 0.13]	− 0.10	[− 0.22, 0.03]	0.01	[− 0.01, 0.03]
COWAT-Semantic	0.16	[0.03, 0.28]	− 0.11	[− 0.22, 0.00]	− 0.03	[− 0.04, 0.00]
COWAT-Phonemic	0.11	[− 0.02, 0.24]	− 0.02	[− 0.15, 0.10]	− 0.03	[− 0.05, − 0.01]
GNG	− 0.06	[− 0.20, 0.07]	0.11	[0.01, 0.22]	0.01	[− 0.01, 0.03]
LNST	0.00	[− 0.13, 0.15]	0.03	[− 0.10, 0.17]	− 0.03	[− 0.05, − 0.01]
Stroop	0.06	[− 0.08, 0.19]	− 0.01	[− 0.12, 0.12]	− 0.01	[− 0.04, 0.01]
WCST	0.02	[− 0.12, 0.16]	0.02	[− 0.10, 0.14]	− 0.01	[− 0.03, 0.02]

*ANT* attention network test, *COWAT* controlled oral word association test, *GNG* go/no-go, *LNST* letter number sequencing task, *WCST* Wisconsin card sorting test, *HDI* highest density interval.

## Discussion

In the present study, we aimed to unravel the influence of familiarity and context on the processing of metaphors with respect to individuals’ executive functions. To this end, we examined metaphor comprehension using the drift–diffusion model, hoping to obtain a better understanding of the detailed processes that underlie metaphor comprehension. Our results demonstrated that familiarity, compared to context, had more substantial impact on the decision process of metaphor comprehension (Figs. 1 and 4). Individuals’ interference control measured by the Semantic fluency task of the Controlled Oral Word Association Test (COWAT-Semantic) was significantly correlated with the processing of metaphors in RTs as well as in drift rates (Figs. 2 and 5). Therefore, we suggest that interference control plays a key role in the decision process of successful metaphor comprehension.

### Impact of interference control on metaphor comprehension

We found a significant negative correlation between RTs and COWAT-Semantic scores (Fig. 2), demonstrating that the higher performance in the COWAT-Semantic test, the lower RTs in metaphor comprehension. Good interference control is known to be responsible for better COWAT performance^61^. During the COWAT, one has to generate words according to a semantic or lexical association while suppressing repeated and/or irrelevant responses, and thus, successful performance in the COWAT requires suppressing interference^62,63^. A study that analyzed the reliability and validity of COWAT scores also proposed that successful COWAT performance necessitates the ability to retrieve words in a non-routine manner while suppressing habitual or prepotent responses^61^. Consequentially, COWAT-Semantic is suitable for assessing individual differences in interference control and, in virtue of the negative correlations between the COWAT performance and RTs in the present study, we suggest that interference control is deeply intertwined with metaphor comprehension, possibly aiding in comprehending metaphorical expressions. Resultingly, participants with higher scores in COWAT-Semantic, having better interference control, were relatively fast in metaphor comprehension.

Furthermore, in the drift–diffusion model, COWAT-Semantic was the only neuropsychological test in which better performance was linked to higher drift rates in metaphor comprehension (Fig. 5). It is important to note that higher drift rates have been known to correspond to individuals’ better performance^9,13,64^. More intriguingly, participants’ higher performance in COWAT-Semantic exhibited shorter non-decision times (Fig. 5). This finding is important as the shorter non-decision time may be influenced by good inhibition of unrelated information, leading to faster processing in metaphor comprehension^65^. All these results denote a beneficial role of good interference control in the processing of metaphors.

The relationship between drift rates and interference control needs further discussion. In previous studies, interpreting drift rates in terms of individual differences has been associated with efficient processing of a given task. This suggests that the higher the drift rate is, the higher the possibility is that people cope with a task more efficiently and more easily. For example, drift rates have been scrutinized to serve as a measure for general cognitive performance^16^, showing that drift rates in an item recognition task were positively correlated with general intelligence. Further compelling evidence was found in a study in which a close relationship between drift rates, working memory, and reasoning was reported^15^. Here, participants were required to complete a set of different choice reaction tasks which showed their drift rates were strongly correlated with their working memory capacity and reasoning ability. A study of attention-deficit hyperactivity disorder (ADHD) patients also asserted that drift rates can be used to serve as a measure for executive functions^66^. Here, children with ADHD performed worse on tasks related to inhibitory control, with lower drift rates compared to children of a control group. Taken together, we suggest that drift rates can be used as a measure of executive functions; more specifically, interference control.

It is worth noting the active role of interference control in various language processes. For example, bilinguals switch between two languages efficiently by taking advantage of contextual cues in the environment to facilitate their linguistic performance as well as by suppressing interference of the language that is currently not in use^67^. Several lines of research also have suggested that response inhibition and interference suppression are responsible for distinguishing behavioral outputs of bilinguals from those of monolinguals^68,69^. Inhibition has also been reported to have leverage on efficient processing of homophones and homonyms^70,71^. These studies indicate that interference control exerts an effect on restraining unnecessary information and choosing apt information in diverse aspects of language processing. Likewise, successful metaphor comprehension requires good interference control to facilitate properties of the metaphor vehicle that are suitable for interpretation while suppressing properties that are irrelevant.

In accordance with our hypothesis, our data showed that the executive functions other than inhibition were not significantly involved during metaphor processing. In light of Miyake’s work^44^, we attempted to scrutinize the degree to which specific abilities are needed for successful metaphor comprehension, using different neuropsychological tests. However, participants’ performance (i.e., RTs and drift rates) in metaphor comprehension tasks were only correlated with scores of the COWAT-Semantic but not with that of the other tests, such as the Attention Network Test (ANT), Go/No-Go (GNG) task, Letter Number Sequencing Task (LNST), Stroop task, and Wisconsin Card Sorting Test (WCST). While much of the data from previous studies has argued that every component of executive functions (i.e., working memory, cognitive flexibility, and inhibition) contribute to metaphor comprehension^30,34,45–49,51–53^, our results suggest that inhibition may be the most relevant executive function engaged in metaphor comprehension.

### Influence of familiarity and context on metaphor comprehension

We have observed increased drift rates in FMs compared to NMs (Fig. 4) in all seven *Full* models. Previous studies showed a negative correlation between task difficulty and drift rates, showing that drift rates increased as task difficulty decreased^9,13,64^. Therefore, FMs may be characterized as being easier to be processed than NMs. This interpretation was also supported by faster RTs and higher accuracies in FMs compared to NMs in the present study (Fig. 1).

Several viewpoints have been addressed regarding what makes the processing of FMs easier than NMs. According to the feature alignment theory^23^, the overlapping features between the topic and vehicle of a metaphor become integrated over time, which makes understanding metaphorical meanings easier^72–74^. The property attribution viewpoint^20,75^ posits an argument that familiar metaphors, being recognized as categorical assertions, are understood as quickly and automatically as literal expressions^20,75^. The career of metaphor hypothesis holds that, in the case of a novel metaphor, people undergo a comparison process, searching for common attributes between the topic and vehicle of the metaphor^23^. Enhancing relevant properties and inhibiting irrelevant ones of the vehicle is also a critical issue of metaphor comprehension^30,31^. A detailed discussion of these theories is beyond the scope of the present study. However, an explanation pertaining to inhibition should be given on why it is easier to process FMs than NMs. It has been shown that retrieving previously integrated metaphorical features is relatively easy in FMs, whereas additional inhibition is needed to filter out irrelevant attributes of the vehicle in NMs^30,76^. More generally, executive functions are known to be more actively involved in processing NMs than in FMs. For example, NMs led to substantial neural activation in frontal brain regions that are known to be involved in executive functions^42,77^. Another study showed that patients with Alzheimer’s disease, specifically having deficits in executive functions, performed poorly in novel metaphor tasks^78^. Taken together, inhibition has been adduced to play an important role in metaphor comprehension, particularly for NMs. However, our results showed that inhibition is closely related to FMs as well as NMs (Fig. 2). This may be derived from possible differences between the present study and previous ones in several aspects such as study designs, measures of inhibition, and participant groups. Therefore, a future study should be conducted with an objective and quantified method to measure the degree of inhibition involved in FMs and NMs to address the potential effects mediated by inhibition in familiar and novel metaphor comprehension.

With respect to context, we could not find a reliable difference in drift rates between SCs and OCs with 95% HDIs including zero (Table 2, Fig. 4), which indicates that contextual information seems to have a marginal influence on metaphor comprehension in the present study. This is at variance with the well-known role of context that has been known to assist the understanding of FMs and NMs by facilitating meaning selection and construction^30,31,51,79,80^, leading to faster and more accurate responses. The discrepancy between the present study and previous ones may be derived from longer but more accurate responses in OCs. The reason participants responded more accurately in OCs than in SCs in both FMs and NMs deserves a comprehensive discussion. This may be due to the increased salience generated in OC when one is presented with features that are semantically incompatible^81^, as in the case of antonyms, for instance. A word and its antonym are generally similar in many aspects, but they differ particularly in one dimension^82,83^. Likewise, the OC sentences of the present study function similarly to antonyms in the way that an attribute delivered from a contextual sentence is opposite from the main feature of the topic used for the NM. For example, when the NM (e.g., “He is glue”) follows the OC (e.g., “He is talented in alienating a friend from others”), the attribute of the vehicle ‘glue’—being sticky and adhesive—denotes the opposite situation described in the context with the word ‘alienating’. Furthermore, since antonyms are known to comprise a large portion of our mental lexicon^81^, OCs may have enabled opposite features to be easily noticeable and accessible to the participants during the metaphor comprehension task. Resultingly, the semantic contrasts created by OCs made metaphor comprehension relatively easy, generating higher accuracies compared to SCs. In this regard, the marginal effect of context in drift rates may be construed by what the drift rate represents. The drift rate is estimated by combining multiple behavioral measures (i.e., RTs and accuracies) simultaneously. As such, the drift rate reflects both RT and accuracy: higher drift rates generate responses with faster RTs and higher accuracies, whereas lower drift rates engender responses with slower RTs and lower accuracies^9^. As mentioned earlier, our data showed slower RTs but higher accuracies in OCs than in SCs, which may have influenced the drift rates to be low in terms of the slow RTs and, at the same time, to be high with respect to the high accuracies. In the end, this interaction between the slow RTs and high accuracies in OCs may have canceled out the effect of drift rates, leaving marginal effects of drift rates between OCs and SCs.

One might call into question whether some of our metaphor sentences, particularly in OCs, may be interpreted as being irony or sarcasm. A crucial distinction between a metaphor and irony is that different comprehension demands are required^84,85^. Since successful comprehension of metaphors is accomplished by comparing the characteristics of the topic and vehicle of the metaphor, knowledge of the two domains (i.e., topic and vehicle) is essential. On the other hand, understanding irony necessitates inferences about the speaker’s intentions^84,85^. In the present study, participants were requested to simply decide whether the metaphor made sense or not after reading the context sentence. Thus, it is unlikely for them to be actively involved in guessing the speaker’s intentions in metaphors. Moreover, we never mentioned to the participants that the experiment is related to either metaphors or irony. Therefore, it is improbable that the participants considered our stimuli to be irony.

Another issue is whether the drift–diffusion model is applicable to data involved in relatively slow cognitive processes such as metaphor comprehension as in the present study. Originally, it was argued that the drift–diffusion model is applicable only to fast RT tasks with mean RTs of maximum 1.5 s per trial^9,86,87^. However, researchers have recently shown that the drift–diffusion model can be also used in modeling slow RT data^88^, suggesting that the model may be widely applicable even in psycholinguistic research^89^. In fact, metaphor comprehension can be either a rapid process with less than 1.5 s^24,90,91^, or a more time-consuming process^46,92^. In our study, we observed that participants completed the metaphor comprehension within 1.03 s on average. Accordingly, we posit that using the drift–diffusion model in our study was suitable for scrutinizing the process of metaphor comprehension.

The present study has some limitations. Firstly, the degree of interpretability between FMs and NMs differed. Although novel metaphors are known to be more difficult to interpret^36,93,94^ than conventional ones, we were unable to differentiate whether the effect of familiarity between FMs and NMs in our study was due to the dissimilar familiarity or interpretability of the metaphors. To tease these two apart, it would be worth controlling for aptness between FMs and NMs—the degree to which a metaphor vehicle captures important features of a metaphor topic^95^—in future studies, because aptness is known to influence interpretability of the metaphor^95^. Secondly, one should consider norming the metaphorical sentences in terms of their suitability with the supporting and opposing contexts, because contextual information shapes the interpretation of metaphors^40^. Thirdly, some of the metaphoric stimuli were chosen from the Standard Korean Dictionary, which means that those words could lose their figurativeness and their meanings could be lexicalized. Lexicalized metaphors may be processed differently from non-lexicalized metaphors in that comprehending lexicalized metaphors is an automatic process, whereas comprehending non-lexicalized metaphors is a controlled process^25,96^. Therefore, the distinction between lexicalized- and non-lexicalized metaphors should be considered in future studies. Fourthly, one should also consider including a proper control condition composed of literal sentences, which would function as a reference to provide a good basis in the understanding of metaphor comprehension in comparison with the experimental condition. Lastly, even if the boundary separation turned out to be related to participants’ performance in the COWAT-Semantic and GNG (Fig. 5), its relationship with metaphor comprehension remains unknown. Boundary separation is the evidence required to make a response; large values indicate that more information needs to be accumulated before a decision is made^13,97,98^. Unfortunately, we were unable to address the relationship between boundary separation and metaphor comprehension in the current study, and thus this requires further investigation.

## Conclusion

By means of fine-grained assessment of individuals’ executive functions and computational modeling using the drift–diffusion model, we have made great strides toward understanding the underlying cognitive processes associated with metaphor comprehension, particularly the influential role of interference control in the processing of metaphor.

## Methods

### Participants

#### Participants in the stimuli norming study

Twenty-two undergraduate or graduate students who did not take part in the metaphor comprehension task participated in the stimuli norming study via an online survey tool (SurveyMonkey Inc., San Mateo, California, USA, www.surveymokey.com) (See Table 4 for demographics). Participants were rewarded 10,000 KRW once they had finished the survey.

**Table 4﻿:** Demographics of participants from the norming study and metaphor comprehension task and their scores of neuropsychological tests (mean ± SD).

	Norming study	Metaphor comprehension task
Age (years)	22.73 ± 2.12	21.21 ± 1.93
Gender (M/F)	12/10	17/21
Years of schooling	14.59 ± 1.26	14.39 ± 1.87
Handedness: LQ	All right-handed (self report)	All right-handed (91.84 ± 10.36)
ANT	n/a	0.20 ± 0.05
COWAT-Semantic		44.79 ± 10.00
COWAT-Phonemic		52.71 ± 13.05
GNG		0.64 ± 0.16
LNST		13.00 ± 2.67
Stroop		0.05 ± 0.02
WCST		5.92 ± 1.05

*LQ* laterality quotient^99^, *ANT* attention network test, *COWAT* controlled oral word association test, *GNG* go/no-go, *LNST* letter number sequencing task, *WCST* Wisconsin card sorting test, *n/a* not applicable.

#### Participants in the metaphor comprehension task

Forty-one native Korean speakers participated in the metaphor comprehension task. Exclusion criteria were color blindness and a history of medical or psychiatric illness. We excluded one participant due to his or her past and ongoing history of psychiatric illness and two participants due to their inappropriate responses in the main experiment (see “Data analysis” for more details). All participants were undergraduate or graduate students. Right handedness was confirmed using the Edinburgh Handedness Inventory^99^. We also used Ishihara plates^100^ to screen for color blindness. All participants were informed about the possibility of being dismissed from the experiment without any disadvantage, signing a written informed consent form accordingly. They were rewarded 15,000 KRW once they had completed the experiment with an overall accuracy of above 70%. A detailed summary of the participants is shown in Table 4. The experiment was conducted in accordance with the recommendations of the Daegu Gyeongbuk Institute of Science and Technology (DGIST) ethics committee and was approved by the DGIST ethics committee in accordance with the Declaration of Helsinki.

### Experimental design and materials

We created stimuli with two factors (FAMILIARITY and CONTEXT) with two levels (familiar metaphor vs. novel metaphor, supporting context vs. opposing context) to examine the influence of the two factors on metaphor comprehension and their interactions with individuals’ different levels of executive functions. The stimulus set consisted of 124 Korean sentence pairs, with the first being a context sentence and the second sentence a metaphor. There were four experimental conditions: a supporting context paired with a familiar metaphor (SC–FM) or a novel metaphor (SC–NM), and an opposing context paired with a familiar metaphor (OC–FM) or a novel metaphor (OC–NM).

We constructed metaphors as the simple form of “X is Y” (see Table 5 for examples) to remove unnecessary processes for sentence comprehension possibly caused by complex syntactic structures. We only used pronouns (i.e., ‘he’ or ‘she’) for the subject “X”, which is referred to as a topic of the metaphor^18^. “Y” is the so-called vehicle of the metaphor, which allows metaphorical reasoning to occur by relating the topic to the vehicle’s notable characteristic^18^. Unlike metaphors, context was constructed without any designated forms. Each metaphor sentence was presented together with either SC or OC to participants. Each participant completed all four conditions in a pseudo-randomized order.

**Table 5:** Examples of context and metaphor sentences.

Condition	Context sentence	Metaphor sentence
SC–FM	She knows almost everything.	She is an encyclopedia.
OC–FM	She lacks basic common sense.	
SC–NM	He arranged many blind dates.	He is glue.
OC–NM	He is talented in alienating a friend.	

*SC–FM* supporting context with familiar metaphor, *OC–FM* opposing context with familiar metaphor, *SC–NM* supporting context with novel metaphor, *OC–NM* opposing context with novel metaphor.

The metaphor sentences were comprised of 62 FMs and 62 NMs. FMs were generated by either choosing words that have figurative meanings from the Standard Korean Dictionary^101^ or selecting words that have been used conventionally as metaphors. Familiarity of these metaphors were later confirmed through a stimulus norming study. NMs were devised with words representing objects, living things, or places that do not have conventional figurative meanings, but still have prominent features such that the intended meanings of newly formulated metaphors were able to be delivered if appropriate contextual information was provided. To validate that our newly made NMs were truly new to people, we had six examiners who were undergraduate or graduate students examine the stimuli and selected expressions that everyone confirmed to be novel.

In addition, we conducted a web-based survey (SurveyMonkey Inc., San Mateo, California, USA, www.surveymokey.com) with 22 new participants to ascertain whether they considered our FMs or NMs to be familiar or new to them, thereby eliminating potentially confounding factors known to affect metaphor processing^20,102–105^ such as familiarity, frequency, interpretability, concreteness, and emotional valence. For each word or metaphor expression, participants were asked to rate the aforementioned five aspects using a seven-point scale. Familiarity was rated to distinguish FMs from NMs (1 for *Very unfamiliar*; 7 for *Very familiar*). Word frequency was measured to verify that all the words used in the metaphor sentences had similar frequency. This was based on the previous result showing that words with low frequency are processed more slowly than those with high frequency^102^. To do this, we asked the participants to report how frequently they encountered the words in their daily lives (1 for *Very rare*; 7 for *Very often*). The interpretability of all the metaphorical expressions, which indicates how easily one can derive a meaning from the expression^103^, were also rated (1 for *Very difficult*; 7 for *Very easy*). Since novel metaphors are known to be more difficult to interpret compared to familiar metaphors^36,93,94^, we expected high interpretability values for familiar metaphors and low values for novel metaphors. Concreteness of the vehicles were investigated based on the previous result, indicating that processing a concrete word is faster than an abstract word^104^ (1 for *Very abstract*; 7 for *Very concrete*). Lastly, positive words are suggested to be processed faster than negative words^105^, and thus we had to ensure that the words used in FMs and NMs had comparable emotional valence (1 for *Very negative*; 7 for *Very positive*). Overall, the words used for metaphors in the present study had balanced values over frequency (FM: mean = 3.75, SEM = 0.11; NM: mean = 3.71, SEM = 0.13), concreteness (FM: mean = 4.57, SEM = 0.10; NM: mean = 4.84, SEM = 0.10), and emotional valence (FM: mean = 3.73, SEM = 0.17; NM: mean = 3.81, SEM = 0.08). As we anticipated, significant differences between the FMs and NMs were found only in familiarity [FM: mean = 4.44, SEM = 0.11; NM: mean = 2.51, SEM = 0.09; *t* (115.83) = 13.49, *p* < 0.001] and interpretability [FM: mean = 4.79, SEM = 0.10; NM: mean = 2.79, SEM = 0.09; *t* (121.21) = 15.19, *p* < 0.001]. Therefore, we verified that the FMs and NMs in the present study were controlled for any unwanted parameters (i.e., frequency, concreteness, and emotional valence).

For the context, 248 sentences were constructed. Half of them were used for SC and the other half for OC. They were paired with 62 FMs and 62 NMs. SC was constructed to aid comprehension of metaphors by increasing the information of the ground that well associates the topic to the vehicle of a metaphor. Conversely, OC was made to hinder metaphor comprehension by providing contradictory information against the ground. SCs and OCs were examined by ten native Koreans and all of them approved that the contexts supported or disrupted the understanding of the ensuing metaphors, respectively.

### Procedures

All participants completed the following seven neuropsychological tests prior to the metaphor comprehension task: Attention Network Test (ANT), Semantic fluency task of Controlled Oral Word Association Test (COWAT-Semantic), Phonemic fluency task of COWAT (COWAT-Phonemic), Go/No-Go (GNG) task, Letter Number Sequencing Task (LNST), Stroop task, and the Wisconsin Card Sorting Test (WCST). These tests enabled us to assess participants’ different cognitive capabilities such that we could investigate the differential contributions of individuals’ executive functions on various metaphor conditions. The details of the neuropsychological tests are given in the “Supplementary Materials”.

The metaphor comprehension task consisted of a short practice session and the main experiment. The practice session was made up of two trials for each condition, which were not used in the main experiment. Throughout the practice and main experiment, a fixation cross was shown for 1 s at the center of a screen. A context sentence was then displayed for 2.5 s, followed by a metaphor sentence being presented until a response was made (maximum duration: 5 s). Participants were required to judge as fast and accurately as possible whether the context and metaphor sentences together made sense or not by pressing the F key or J key on a keyboard. Key distribution for the yes or no responses was counterbalanced across participants. The time lapsing from the appearance of the metaphor sentence until key press was recorded as RT and the percentage of correct responses for each condition was quantified as accuracy. The experiment was conducted using PsychoPy software in Python, Version 1.85.2^106^.

### Data analysis

Two out of the 40 participants were excluded from the analysis whose average RTs of the metaphor comprehension task or average scores of the neuropsychological tests were more than three standard deviations away from the mean across participants. This resulted in disposal of 5% of the total data (248 from 4960 responses). In addition, we removed outlier RTs that were beyond three standard deviations from the mean for each participant, which led to disposal of 10.44% of the remaining data (492 from 4712 responses). We conducted two-way repeated measures analyses of variance (ANOVA) using the RTs and accuracies of the metaphor comprehension task, with factors FAMILIARITY (FM and NM) and CONTEXT (SC and OC). The ez package from R software was used for the analyses^107^. We included only correct responses for the RT analyses.

We obtained Pearson correlation coefficients between the scores of the seven neuropsychological tests and the RT data from the four conditions of the metaphor comprehension task to examine the roles of different executive functions on metaphor comprehension and to see how individual differences in executive functions affect metaphor processing. Here, we converted participants’ mean RT data and their neuropsychological test scores into z-scores to allow comparison of data from different distributions.

### Modeling behavioral data

#### Drift–diffusion model

The drift–diffusion model, as one of the sequential sampling models, assumes that a decision is made once it reaches a decision boundary while information is continuously accumulated^9,11^. Using RTs and accuracies simultaneously, the drift–diffusion model separates a decision process into four main parameters such as drift rate ($v$), decision boundary ($a$), non-decision time ($t$), and starting point ($z$), along with three additional parameters accounting for inter-trial variability of drift rate (${s}_{v}$), non-decision time (${s}_{t}$), and starting bias (${s}_{z}$). We adopted this model to explain latent processes underlying metaphor comprehension, which would be hard to explain with behavioral outputs alone.

The diffusion model parameters were estimated using the hierarchical drift–diffusion model (HDDM) package^108^ written in Python, which analyzes behavioral data using the Bayesian hierarchical model. It assumes that each participant’s model parameters are sampled from population-level distributions, which shrinks the individuals’ parameters to be closer to the population mean. Thus, the HDDM provides reliable estimates of individuals’ parameters when the number of observations from each participant is relatively small^109^. Also, the Markov Chain Monte Carlo technique used for estimating parameters in the HDDM package gives a full posterior distribution of each parameter rather than just point estimates, and thus one can directly test a hypothesis on the posterior distribution of parameters^110^. In the present study, since our main research question refers to the differences between experimental conditions on the population level, we applied all our tests to the population-level posterior distribution of parameters accordingly.

#### Model specification

We allowed the drift rate to vary across FAMILIARITY (FM and NM) and CONTEXT (SC and OC), because these were the two key factors that were expected to influence participants’ responses. In addition, we hypothesized that the drift rate ($v$), decision boundary ($a$), and non-decision time ($t$) would be affected by individuals’ differences in executive functions that were represented by the scores of seven neuropsychological tests. Resultingly, seven *Full* models, one for each of the seven neuropsychological tests, incorporated one continuous predictor variable (participants’ neuropsychological scores) into each of the three decision parameters accounting for individual differences (drift rate, decision boundary, and non-decision time). With this approach, we intended to clearly differentiate the effect of each test and to avoid the arbitrary and complex interactions that could have occurred if we had considered all the tests simultaneously. Decision boundary ($a$) and non-decision time ($t$) were fixed within participants across the conditions, and accordingly, a participant’s response differences between conditions could only be captured by drift rates^109^. Inter-trial variability was considered in drift rate (${s}_{v}$) and non-decision time (${s}_{t}$). In addition, correct and incorrect responses were mapped as upper boundaries and lower boundaries, respectively. We therefore fixed starting point ($z$) at 0.5 to prevent a bias since we did not expect a biased response in the setting of correct/incorrect boundaries.

Informed priors were applied for each parameter to be inferred in a moderate range based on previous survey parameter values^97^. In the estimation process, we generated 12,000 samples using a Markov chain Monte Carlo algorithm^111^, including 2000 burn-in samples which were later discarded to prevent the effect of initial exploratory values before convergence. We used the Geweke statistic^112^ to ensure that chains properly converged. Posterior predictive checks were performed along with visual inspections to examine whether predicted data followed observed RT distribution or not.

To validate our model specification, we performed a model comparison with two additional models. One was a *Null* model that assumed all the parameters to be fixed between conditions and ignored individual differences in the scores of seven neuropsychological tests. The other was an *FC* model (*familiarity and context*), assuming that only trial-type altered the decision process. Therefore, drift rates varied according to the two factors—FAMILIARITY and CONTEXT—while individual differences in neuropsychological tests were not considered. We used the deviance information criterion (DIC), which is a measure to assess model fit in hierarchical models^59^ so that we could compare different models in the current study to show that our suggested models outperformed two additional models.

### Statistical analyses

All the analyses were tested directly on the population-level posterior estimates of *Full* models. This is a common practice when a research question is focused on comparing different groups rather than individual-level parameters^60,98^. On the basis of a Bayesian hypothesis testing perspective, it is reliable to say that a factor has an effect on the drift rate when 95% highest density interval (HDI) of the estimated effect does not include zero. Therefore, we calculated 95% HDIs for our parameters of interest and set them as decision criteria to indicate whether zero was included or not^60^. Effects of familiarity and context on metaphor comprehension were tested using drift rates in this regard. For example, the effect of COWAT-Semantic on drift rates of metaphor comprehension was studied by testing whether 95% HDI of the population-level distribution for drift rates moved away from zero or not. Additionally, relations between individual differences in neuropsychological tests and estimated decision parameters (drift rates, boundary separation, non-decision time) were tested using 95% HDI.

## Supplementary Information

Supplementary Information.

## References

[1] Richards, I. A. & Lewis, C. S. *The Philosophy of Rhetoric**.* (Oxford University Press, 1936).

[2] Grice, H. P. Logic and conversation. in *Syntax and semantics 3, Speech acts* (eds. Cole, P. & Morgan, J. L.) 41–58 (Academic Press, 1975).

[3] Bohrn IC, Altmann U, Jacobs AM (2012). Looking at the brains behind figurative language—A quantitative meta-analysis of neuroimaging studies on metaphor, idiom, and irony processing. Neuropsychologia.

[4] Amodio DM, Frith CD (2006). Meeting of minds: The medial frontal cortex and social cognition. Nat. Rev. Neurosci..

[5] Blasko DG, Briihl DS (1997). Reading and recall of metaphorical sentences: Effects of familiarity and context. Metaphor. Symb..

[6] Pexman PM, Ferretti TR, Katz AN (2000). Discourse factors that influence online reading of metaphor and irony. Discourse Process..

[7] Holyoak KJ, Stamenković D (2018). Metaphor comprehension: A critical review of theories and evidence. Psychol. Bull..

[8] Ratcliff R (2002). A diffusion model account of response time and accuracy in a brightness discrimination task: Fitting real data and failing to fit fake but plausible data. Psychon. Bull. Rev..

[9] Ratcliff R, McKoon G (2008). The diffusion decision model: Theory and data for two-choice decision tasks. Neural Comput..

[10] Ratcliff R, Rouder JN (1998). Modeling response times for two-choice decisions. Psychol. Sci..

[11] Ratcliff R, Rouder JN (2000). A diffusion model account of masking in two-choice letter identification. J. Exp. Psychol. Hum. Percept. Perform..

[12] Farrell, S. & Lewandowsky, S. *Computational modeling of cognition and behavior.* (Cambridge University Press, 2018).

[13] Voss A, Rothermund K, Voss J (2004). Interpreting the parameters of the diffusion model: An empirical validation. Mem. Cognit..

[14] Sewell DK, Lilburn SD, Smith PL (2016). Object selection costs in visual working memory: A diffusion model analysis of the focus of attention. J. Exp. Psychol. Learn. Mem. Cogn..

[15] Schmiedek F, Oberauer K, Wilhelm O, Süß H-M, Wittmann WW (2007). Individual differences in components of reaction time distributions and their relations to working memory and intelligence. J. Exp. Psychol. Gen..

[16] Ratcliff R, Thapar A, McKoon G (2011). Effects of aging and IQ on item and associative memory. J. Exp. Psychol. Gen..

[17] Liebman, E., White, C. N. & Stone, P. On the impact of music on decision making in cooperative tasks. in *Proceedings of the 19th international society for music information retrieval conference, ISMIR 2018, paris, france, september 23-27, 2018* (eds. Gómez, E., Hu, X., Humphrey, E. & Benetos, E.) 695–701 (2018).

[18] Lakoff G, Johnson M (2008). Metaphors We Live By.

[19] Holyoak KJ, Stamenkovic D (2018). Metaphor comprehension: A critical review of theories and evidence. Psychol Bull.

[20] Glucksberg S (2003). The psycholinguistics of metaphor. Trends Cogn. Sci..

[21] Giora R (1997). Understanding figurative and literal language: The graded salience hypothesis. Cognitive Linguistics.

[22] Giora R (2002). Literal vs. figurative language: Different or equal?. J. Pragmat..

[23] Bowdle BF, Gentner D (2005). The career of metaphor. Psychol. Rev..

[24] Blasko DG, Connine CM (1993). Effects of familiarity and aptness on metaphor processing. J. Exp. Psychol. Learn. Mem. Cogn..

[25] Blank GD (1988). Metaphors in the lexicon. Metaphor. Symb..

[26] Onishi KH, Murphy GL (1993). Metaphoric reference: When metaphors are not understood as easily as literal expressions. Mem. Cognit..

[27] Giora R, Fein O (1999). On understanding familiar and less-familiar figurative language. J. Pragmat..

[28] Searle, J. R. *Expression and meaning: Studies in the theory of speech acts.* (Cambridge University Press, 1979).

[29] Cardillo ER, Watson CE, Schmidt GL, Kranjec A, Chatterjee A (2012). From novel to familiar: Tuning the brain for metaphors. Neuroimage.

[30] Rubio Fernandez P (2007). Suppression in metaphor interpretation: Differences between meaning selection and meaning construction. J. Semant..

[31] Carston R (2002). Metaphor, ad hoc concepts and word meaning—More questions than answers. UCL Working Papers Linguistics.

[32] Gentner D, Wolff P (1997). Alignment in the processing of metaphor. J. Mem. Lang..

[33] Al-Azary H, Buchanan L (2017). Novel metaphor comprehension: Semantic neighbourhood density interacts with concreteness. Mem. Cognit..

[34] Prat CS, Mason RA, Just MA (2012). An fMRI investigation of analogical mapping in metaphor comprehension: The influence of context and individual cognitive capacities on processing demands. J. Exp. Psychol. Learn. Memory Cognit..

[35] Shinjo, M. & Myers, J. L. The role of context in metaphor comprehension. **26**, 226–241. 10.1016/0749-596x(87)90125-2 (1987).

[36] Carriedo N (2016). The development of metaphor comprehension and its relationship with relational verbal reasoning and executive function. PLoS ONE.

[37] Foss DJ, Jenkins CM (1973). Some effects of context on the comprehension of ambiguous sentences. J. Verbal Learn. Verbal Behav..

[38] Gibbs RW, Gerrig RJ (1989). How context makes metaphor comprehension seem'special'. Metaphor. Symb..

[39] Pickering MJ, Frisson S (2001). Processing ambiguous verbs: Evidence from eye movements. J. Exp. Psychol. Learn. Mem. Cogn..

[40] Ortony A, Schallert DL, Reynolds RE, Antos SJ (1978). Interpreting metaphors and idioms: Some effects of context on comprehension. J. Verbal Learn. Verbal Behav..

[41] Gildea P, Glucksberg S (1983). On understanding metaphor: The role of context. J. Mem. Lang..

[42] Columbus G (2014). Individual differences in executive control relate to metaphor processing: An eye movement study of sentence reading. Front. Hum. Neurosci..

[43] Yang FG, Edens J, Simpson C, Krawczyk DC (2009). Differences in task demands influence the hemispheric lateralization and neural correlates of metaphor. Brain Lang..

[44] Miyake A (2000). The unity and diversity of executive functions and their contributions to complex "Frontal Lobe" tasks: A latent variable analysis. Cogn. Psychol..

[45] Kazmerski VA, Blasko DG, Dessalegn BG (2003). ERP and behavioral evidence of individual differences in metaphor comprehension. Mem. Cognit..

[46] Chiappe DL, Chiappe P (2007). The role of working memory in metaphor production and comprehension. J. Mem. Lang..

[47] Blasko DG (1999). Only the tip of the iceberg: Who understands what about metaphor?. J. Pragmat..

[48] Mashal N, Kasirer A (2011). Thinking maps enhance metaphoric competence in children with autism and learning disabilities. Res. Dev. Disabil..

[49] Diamond A (2013). Executive functions. Annu. Rev. Psychol..

[50] Lehto JE, Juujärvi P, Kooistra L, Pulkkinen L (2003). Dimensions of executive functioning: Evidence from children. Br. J. Dev. Psychol..

[51] Recanati F (1995). The alleged priority of literal interpretation. Cogn. Sci..

[52] Gernsbacher MA, Keysar B, Robertson RR, Werner NK (2001). The role of suppression and enhancement in understanding metaphors. J. Mem. Lang..

[53] Pierce RS, Maclaren R, Chiappe DL (2010). The role of working memory in the metaphor interference effect. Psychon. Bull. Rev..

[54] Orellana, G. & Slachevsky, A. Executive functioning in schizophrenia. *Front. Psychiatry* **4**10.3389/fpsyt.2013.00035 (2013). 10.3389/fpsyt.2013.00035 PMC3690455 23805107

[55] Mashal N, Vishne T, Laor N, Titone D (2013). Enhanced left frontal involvement during novel metaphor comprehension in schizophrenia: Evidence from functional neuroimaging. Brain Lang..

[56] Nigg JT (2000). On inhibition/disinhibition in developmental psychopathology: Views from cognitive and personality psychology and a working inhibition taxonomy. Psychol. Bull..

[57] Glucksberg S, Newsome MR, Goldvarg Y (2001). Inhibition of the literal: Filtering metaphor-irrelevant information during metaphor comprehension. Metaphor. Symb..

[58] Simpson, J. A. & Weiner, E. S. C. *The Oxford English dictionary.* (Oxford University Press, 1989).

[59] Spiegelhalter DJ, Best NG, Carlin BP, Van Der Linde A (2002). Bayesian measures of model complexity and fit. J. Roy. Stat. Soc. Ser. B. (Stat. Method.).

[60] Kruschke JK (2013). Bayesian estimation supersedes the t test. J. Exp. Psychol. Gen..

[61] Ross T (2007). The reliability and validity of qualitative scores for the Controlled Oral Word Association Test. Arch. Clin. Neuropsychol..

[62] Perret E (1974). The left frontal lobe of man and the suppression of habitual responses in verbal categorical behaviour. Neuropsychologia.

[63] Collins AM, Loftus EF (1975). A spreading-activation theory of semantic processing. Psychol. Rev..

[64] Philiastides MG, Ratcliff R, Sajda P (2006). Neural representation of task difficulty and decision making during perceptual categorization: A timing diagram. J. Neurosci..

[65] Ratcliff R, Smith PL, Brown SD, McKoon G (2016). Diffusion decision model: Current issues and history. Trends Cogn. Sci..

[66] Karalunas SL, Huang-Pollock CL (2013). Integrating impairments in reaction time and executive function using a diffusion model framework. J. Abnorm. Child. Psychol..

[67] Luk G, Anderson JA, Craik FI, Grady C, Bialystok E (2010). Distinct neural correlates for two types of inhibition in bilinguals: Response inhibition versus interference suppression. Brain Cogn..

[68] Bialystok E, Martin MM (2004). Attention and inhibition in bilingual children: Evidence from the dimensional change card sort task. Dev. Sci..

[69] Bialystok E (2006). Effect of bilingualism and computer video game experience on the Simon task. Can. J. Exp. Psychol./Revue canadienne de psychologie expérimentale.

[70] Zhang-Yaxu W-L (2003). Inhibitory processes in the recognition of homophone meanings in Chinese. Acta Psychol. Sin..

[71] Jones JL (1989). Multiple access of homonym meanings: An artifact of backward priming?. J. Psycholinguist. Res..

[72] Goldstein A, Arzouan Y, Faust M (2012). Killing a novel metaphor and reviving a dead one: ERP correlates of metaphor conventionalization. Brain Lang..

[73] Mashal N (2013). The role of working memory in the comprehension of unfamiliar and familiar metaphors. Lang. Cogn..

[74] Lai VT, Curran T (2013). ERP evidence for conceptual mappings and comparison processes during the comprehension of conventional and novel metaphors. Brain Lang..

[75] Glucksberg S, McGlone MS, Grodzinsky Y, Amunts K (2001). Understanding Figurative Language: From Metaphor to Idioms.

[76] Glucksberg, S., Manfredi, D. A. & McGlone, M. S. Metaphor comprehension: How metaphors create new categories. in *Creative thought: an investigation of conceptual structures and processes* (eds. Ward, T. B., Smith, S. M. & Vaid, J.) 327–350 (American Psychological Association, 1997).

[77] Mashal N, Faust M, Hendler T, Jung-Beeman M (2007). An fMRI investigation of the neural correlates underlying the processing of novel metaphoric expressions. Brain Lang.

[78] Amanzio M, Geminiani G, Leotta D, Cappa S (2008). Metaphor comprehension in Alzheimer’s disease: Novelty matters. Brain Lang..

[79] Posner, M. I. & Snyder, C. R. R. Attention and Cognitive Control. in *Cognitive psychology: Key readings *(eds. Balota, D. A. & Marsh, E. J.) 205–223 (Psychology Press, 2004).

[80] Neill WT, Valdes LA, Terry KM (1995). Interference and Inhibition in Cognition.

[81] Hutchison KA (2003). Is semantic priming due to association strength or feature overlap? A microanalytic review. Psychon. Bull. Rev..

[82] Cruse DA, Cruse DA (1986). Lexical Semantics.

[83] Jeon HA, Lee KM, Kim YB, Cho ZH (2009). Neural substrates of semantic relationships: Common and distinct left-frontal activities for generation of synonyms vs. antonyms. Neuroimage.

[84] Winner E, Gardner H (1993). Metaphor and irony: Two levels of understanding. Metaphor Thought.

[85] Colston HL, Gibbs RW (2002). Are irony and metaphor understood differently?. Metaphor. Symb..

[86] Ratcliff R, Frank MJ (2012). Reinforcement-based decision making in corticostriatal circuits: Mutual constraints by neurocomputational and diffusion models. Neural Comput..

[87] Ratcliff R, Thapar A, Gomez P, McKoon G (2004). A diffusion model analysis of the effects of aging in the lexical-decision task. Psychol. Aging.

[88] Lerche V, Voss A (2019). Experimental validation of the diffusion model based on a slow response time paradigm. Psychol. Res..

[89] Lerche V, Christmann U, Voss A (2018). Impact of context information on metaphor elaboration. Exp. Psychol..

[90] Schmidt GL, DeBuse CJ, Seger CA (2007). Right hemisphere metaphor processing? Characterizing the lateralization of semantic processes. Brain Lang..

[91] Bambini V, Bertini C, Schaeken W, Stella A, Di Russo F (2016). Disentangling metaphor from context: An ERP study. Front. Psychol..

[92] Mashal N, Faust M, Hendler T, Jung-Beeman M (2009). An fMRI study of processing novel metaphoric sentences. Laterality.

[93] Lai VT, Curran T, Menn L (2009). Comprehending conventional and novel metaphors: An ERP study. Brain Res..

[94] Arzouan Y, Goldstein A, Faust M (2007). Dynamics of hemispheric activity during metaphor comprehension: electrophysiological measures. Neuroimage.

[95] Thibodeau PH, Durgin FH (2011). Metaphor aptness and conventionality: A processing fluency account. Metaphor. Symb..

[96] Schneider W, Shiffrin RM (1977). Controlled and automatic human information processing: I. Detection, search, and attention. Psychol. Rev..

[97] Matzke D, Wagenmakers EJ (2009). Psychological interpretation of the ex-Gaussian and shifted Wald parameters: A diffusion model analysis. Psychon. Bull. Rev..

[98] Vandekerckhove J, Tuerlinckx F, Lee MD (2011). Hierarchical diffusion models for two-choice response times. Psychol. Methods.

[99] Oldfield RC (1971). The assessment and analysis of handedness: The Edinburgh inventory. Neuropsychologia.

[100] Ishihara S (1972). The Series of Plates Designed as a Test for Colour-Blindess [sic]... 38 Plates Edition.

[101] National Institute of Korean Language. *Standard Korean language dictionary* https://stdict.korean.go.kr (2008).

[102] Gernsbacher MA (1984). Resolving 20 years of inconsistent interactions between lexical familiarity and orthography, concreteness, and polysemy. J. Exp. Psychol. Gen..

[103] Cardillo ER, Schmidt GL, Kranjec A, Chatterjee A (2010). Stimulus design is an obstacle course: 560 matched literal and metaphorical sentences for testing neural hypotheses about metaphor. Behav. Res. Methods.

[104] Binder JR, Westbury CF, McKiernan KA, Possing ET, Medler DA (2005). Distinct brain systems for processing concrete and abstract concepts. J. Cogn. Neurosci..

[105] Kuchinke L (2005). Incidental effects of emotional valence in single word processing: an fMRI study. Neuroimage.

[106] Peirce JW (2007). PsychoPy—Psychophysics software in Python. J. Neurosci. Methods.

[107] Lawrence, M. A. ez: Easy analysis and visualization of factorial experiments. *R Package Version 4.4-0 *(2016).

[108] Wiecki T, Sofer I, Frank M (2013). HDDM: Hierarchical Bayesian estimation of the Drift-Diffusion Model in Python. Front. Neuroinform..

[109] Ratcliff R, Childers R (2015). Individual differences and fitting methods for the two-choice diffusion model of decision making. Decision.

[110] Gelman A (2013). Bayesian Data Analysis.

[111] Gamerman D, Lopes HF (2006). Markov Chain Monte Carlo: Stochastic Simulation for Bayesian Inference.

[112] Geweke J (1992). Evaluating the accuracy of sampling-based approaches to the calculations of posterior moments. Bayesian Stat..

