# Supplementary material for: The critical role of interference control in metaphor comprehension evidenced by the drift–diffusion model
Source: Sci Rep. 2021 Sep 29;11:19292. doi: 10.1038/s41598-021-98351-8 (PMC8481255; doi:10.1038/s41598-021-98351-8)
Supplement: Supplementary file 1 — Supplementary Information. [file 41598_2021_98351_MOESM1_ESM.pdf]

Supplementary Materials

The critical role of interference control in metaphor comprehension evidenced by the drift-diffusion model

Hee-Dong Yoon[1,2,†], Minho Shin[1,†], and Hyeon-Ae Jeon[1,3*]

[1]Department of Brain and Cognitive Sciences, Daegu Gyeongbuk Institute of Science and Technology (DGIST), Daegu, Korea
[2] Convergence Research Advance Center for Olfaction, Daegu Gyeongbuk Institute of Science and Technology (DGIST), Daegu, Korea
[3]Partner Group of the Max Planck Institute for Human Cognitive and Brain Sciences at the Department of Brain and Cognitive Sciences, DGIST, Daegu, Korea

[†]These authors contributed equally to this work.

* Corresponding author

E-mail: jeonha@dgist.ac.kr (HAJ)

1. ***Attention Network Test (ANT)*** The ANT encompasses aspects of the cued reaction time task[1] and the flanker task[2]. Participants' alertness, orientation, and executive attention is assessed[3] in ANT, where they are asked to respond to the direction of only the centrally presented arrow, simultaneously ignoring surrounding stimuli that either point in the same direction (congruent trial) or the opposite direction (incongruent trial) of the center arrow. Responses are known to be slower for incongruent trials compared to congruent trials, since irrelevant information has to be inhibited[2]. In neutral trials, flanking arrows are not displayed, and only the center arrow is shown. At the beginning of each trial, a fixation cross is presented for a random duration between 0.4s and 1.6s. Then a warning sign appears for 0.1s followed by a fixation with an inter-stimulus interval of 0.4s. Arrow stimuli are displayed until a keyboard response is made (maximum duration: 1.7s). The four warning conditions are presented to measure alertness and/or orientation: no cue, center cue, double cue, and spatial cue[3]. For the no-cue conditions, only the fixation cross is presented. For the center cue conditions, participants see an asterisk instead of a fixation cross at the location of the fixation cross. For the double-cue conditions, two warning signs (i.e., asterisks) are shown in the two possible target positions. Lastly, the spatial cues specify where the arrows will appear. Consequently, only the spatial cues provide a valid indication of the target location. Altogether, we focused on the RT differences between congruent and incongruent trials. Only the correct responses were taken into consideration.

2. ***Controlled Oral Word Association Test (COWAT)*** The COWAT measures verbal fluency[4,5]. This test evaluates not only spontaneous generation of words that pertains to a specific category (e.g., "names of animals") within a designated time limit[6] but also inhibition of unnecessary information that is not related to that category[7]. COWAT is

tested with semantic fluency (COWAT-Semantic) and phonemic fluency (COWAT-Phonemic)[8].

2-1. In the COWAT-Semantic, participants are required to name as many words as possible that belong to a given category (e.g., animals) for 60s. This procedure is repeated twice, each time using a different category (i.e., animal names and items found in a grocery store). Participants' performance for the COWAT-Semantic is measured by counting the sum of appropriate answers from the two categories.

2-2. In the COWAT-Phonetic, participants are given 60s to generate as many words as possible that begin with a given consonant. Because our participants were all native Korean speakers, we used Korean consonants (ㄱ, ㅇ, ㅅ)[9]. COWAT-Phonemic performance is calculated by adding the total number of valid responses for all three consonants.

3. ***Go/no-go (GNG) task*** The GNG task measures lack of inhibition[10]. Participants are required to press a button in response to target stimuli and to stop responding when non-target stimuli are presented. We modified Bezdian's[10] computerized design of the task. In our GNG task, the letter (P or R) is used as either a go sign or a no-go sign. When either one of the letters appear on one of the quadrants of the computer screen, participants are requested to press the designated key in response to the go sign and not to react to the no-go signal. The ratio of the go and no-go signs is set as 8:2. There are two sessions, with each session having 160 trials. The go or no-go letter (P or R) is switched between the sessions. Throughout the task, go and no-go signs are presented for 0.5s, and the inter-stimulus interval is set as 0.5s. The accuracy of the no-go trials is used as the GNG task score.

4. ***Letter Number Sequencing (LNS) task*** The LNS task is a subset of the working memory test of the Wechsler Adult Intelligence Scale Version IV[11]. Examiners read out a sequence mixed with numbers and letters to the participants. Participants are asked to respond first by reporting the numbers in order from the smallest number to the largest number, and then letters in alphabetical order. For example, if the participants hear a sequence such as “7, N, 4, L,” their correct answer would be “4, 7, L, N.” The task begins with a two-item trial, with one item increasing every three trials. Participants earn one score for every correct trial, resulting in 24 for the perfect score.

5. ***Stroop task*** The Stroop task is for assessing the ability to inhibit cognitive interference[12]. Participants are presented with names of colors displayed in different font colors and required to name the font color of the words, not to read the words. There are two types of conditions in the task[12,13]: the congruent and incongruent conditions. In the congruent condition, the color name matches the meaning of the word (e.g., a word ‘blue’ printed in blue ink). In the incongruent condition, the color name is different from the word meaning (e.g., the word ‘blue’ printed in red ink). In the present study, we used a computerized version of the Stroop task[14,15]. Stimulus presentation and response acquisition were conducted with Psychophysics Toolbox extensions of the MATLAB software. RTs of the incongruent trials are generally longer than those of the congruent trials, because participants are required to inhibit their automatic responses, that is, reading words as they are written[16]. We recorded each participant’s verbal responses and measured the duration from stimulus presentation until participants responded. The difference in reaction times (RTs) between incongruent and congruent conditions was

calculated as the Stroop score. Only the correct responses were considered for the analysis.

6. ***Wisconsin Card Sorting Test (WCST)*** The ability of set-shifting was assessed by using the WCST[17-20]. Unlike the original version of the test[18], we used a computerized version via PsyToolkit. Four cards were presented on the top position of a monitor screen, and another card was presented at the bottom. Participants were asked to classify the card at the bottom based on the three criteria (classification rules) such as color, shape, or number of symbols and to match it to one of the four cards in the upper card deck with a mouse click. They were provided with feedback in every card selection, which helped them to learn the classification rule. The rule changed every 10 cards, but participants were not aware of the change and had to discover the rule changes themselves[21]. When participants adhered to a previous rule and failed in the adjustment to a new rule, this was considered to be a perseverative error. The number of perseverative errors was counted as the WCST score.

References

1 Posner, M. I. Orienting of attention. *Quarterly Journal of Experimental Psychology* **32**, 3-25 (1980).

2 Eriksen, B. A. & Eriksen, C. W. Effects of noise letters upon the identification of a target letter in a nonsearch task. *Perception & Psychophysics* **16**, 143-149 (1974).

3 Fan, J., McCandliss, B. D., Sommer, T., Raz, A. & Posner, M. I. Testing the efficiency and independence of attentional networks. *Journal of Cognitive Neuroscience* **14**, 340-347, doi:10.1162/089892902317361886 (2002).

4 Butler, M., Retzlaff, P. D. & Vanderploeg, R. Neuropsychological test usage. *Professional psychology: Research and practice* **22**, 510 (1991).

5 Rabin, L. A., Barr, W. B. & Burton, L. A. Assessment practices of clinical neuropsychologists in the United States and Canada: A survey of INS, NAN, and APA Division 40 members. *Archives of Clinical Neuropsychology* **20**, 33-65 (2005).

6 Strauss, E., Sherman, E. M. & Spreen, O. *A compendium of neuropsychological tests: Administration, norms, and commentary*. (American Chemical Society, 2006).

7 Ross, T. P. *et al.* The reliability and validity of qualitative scores for the Controlled Oral Word Association Test. *Archives of Clinical Neuropsychology* **22**, 475-488 (2007).

8 Patterson, J. in *Encyclopedia of Clinical Neuropsychology* (eds Jeffrey S. Kreutzer, John DeLuca, & Bruce Caplan) 703-706 (Springer New York, 2011).

9 Kang, Y. W., Chin, J. H., Na, D. L., Lee, J. H. & Park, J. S. A normative study of the Korean version of Controlled Oral Word Association Test (COWAT) in the elderly. *Korean Journal of Clinical Psychology* **19**, 385-392 (2000).

10 Bezdjian, S., Baker, L. A., Lozano, D. I. & Raine, A. Assessing inattention and impulsivity in children during the Go/NoGo task. *British Journal of Developmental Psychology* **27**, 365-383, doi:10.1348/026151008X314919 (2009).

11 Wechsler, D. *Wechsler Adult Intelligence Scale–Fourth Edition (WAIS–IV).* (San Antonio, TX: The Psychological Corporation, 2008).

12 Stroop, J. R. Studies of interference in serial verbal reactions. *Journal of experimental psychology* **18**, 643 (1935).

13 MacLeod, C. M. Half a century of research on the Stroop effect: an integrative review. *Psychological bulletin* **109**, 163 (1991).

14 Afsaneh, Z. *et al.* Assessment of Selective Attention with CSCWT (Computerized Stroop Color-Word Test) among Children and Adults. *Online Submission* (2012).

15 Penner, I.-K. *et al.* The Stroop task: comparison between the original paradigm and computerized versions in children and adults. *The Clinical Neuropsychologist* **26**, 1142-1153 (2012).

16 Diamond, A. Executive functions. *Annual Review of Psychology* **64**, 135-168, doi:10.1146/annurev-psych-113011-143750 (2013).

17 Grant, D. A. & Berg, E. A behavioral analysis of degree of reinforcement and ease of shifting to new responses in a Weigl-type card-sorting problem. *Journal of experimental psychology* **38**, 404 (1948).

18 Puente, A. Wisconsin card sorting test. *Test critiques* **4**, 677-682 (1985).

19 Miyake, A. *et al.* The unity and diversity of executive functions and their contributions to complex "Frontal Lobe" tasks: a latent variable analysis. *Cogn Psychol* **41**, 49-100, doi:10.1006/cogp.1999.0734 (2000).

20 Diamond, A. Executive functions. *Annu Rev Psychol* **64**, 135-168, doi:10.1146/annurev-psych-113011-143750 (2013).

21 Lysaker, P., Bell, M. & Beam-Goulet, J. Wisconsin Card Sorting Test and work performance in schizophrenia. *Psychiatry Research* **56**, 45-51 (1995).
